# Supplementary material for: Tracking and Controlling Monolayer Water in Gold Nanogaps using Extreme Plasmonic Spectroscopy
Source: Small. 2025 Oct 25;21(49):e07013. doi: 10.1002/smll.202507013 (PMC12696788; doi:10.1002/smll.202507013)
Supplement: Supplementary file 1 — Supporting Information [file SMLL-21-e07013-s001.pdf]

NANO · MICRO
small

Supporting Information

for *Small*, DOI 10.1002/smll.202507013

Tracking and Controlling Monolayer Water in Gold Nanogaps using Extreme Plasmonic Spectroscopy

*Elle W. Wyatt, Sarah May Sibug-Torres, Rakesh Arul, Marika Niihori, Tabitha Jones, James W. Beattie, Bart de Nijs and Jeremy J. Baumberg**

Supplementary Information

Tracking and controlling monolayer water in gold nanogaps using extreme plasmonic spectroscopy

*Elle W. Wyatt, Sarah May Sibug-Torres, Rakesh Arul, Marika Niihori, Tabitha Jones, James W. Beattie, Bart de Nijs, Jeremy J. Baumberg**

E.W. Wyatt, S.M. Sibug-Torres, R. Arul, M. Niihori, T. Jones, J.W. Beattie, J.J. Baumberg
NanoPhotonics Centre, Cavendish Laboratory, Department of Physics, JJ Thompson Avenue, University of Cambridge, Cambridge, CB3 0US, United Kingdom
E-mail: jjb12@cam.ac.uk

B. de Nijs
Physics for Sustainable Chemistry Group, Department of Physics, JJ Thompson Avenue, University of Cambridge, Cambridge, CB3 0US, United Kingdom

Contents:

Supporting text (Supp. Note 1-6)

Figures S1 to S20

Figure S1. TEM images of a free-standing MLagg
Figure S2. AFM of a MLagg prepared with 80 nm AuNP
Figure S3. SERS and Dark field (DF) scattering of a MLagg prepared with 80 nm AuNP
Figure S4. MLagg low and high wavenumber SERS in air, water and DMSO
Figure S5. Repeat SERS data for MLaggs in air, water and DMSO
Figure S6. CB[n] scaffolded MLaggs SERS in air, water and DMSO
Figure S7. Raman of water and DMSO
Figure S8. High and low wavenumber SERS of MLaggs scaffolded in H_2O and D_2O solutions
Figure S9. Repeat SERS data for MLaggs in scaffolded in aqueous and deuterated solutions.
Figure S10. Experimental set-ups for MLagg drying study
Figure S11. Changes in C-H peaks on drying
Figure S12. DFT of CB[5] C-H lines
Figure S13. Heating and cooling
Figure S14. Ratio of CB[5] to Cl^-
Figure S15. Experimental set-up for EC-SERS measurements
Figure S16. Repeat data for EC-SERS measurements.
Figure S17. High and low wavenumber EC-SERS
Figure 18. High and low wavenumber EC-SERS with CB[7] and BPT scaffolds
Figure S19. O-H and O-D in different samples
Figure S20. SERS in high HCl concentrations
Figure S21. C-H in different samples

Tables S1

Table S1. Comparison of experimental studies on water at interfaces.

SI References

Table S1. Comparison of experimental studies on water at interfaces. Where available, details of the SERS substrate, measurement techniques and experimental conditions are included.

**Study**	**Substrate**	**Measurement technique**	**Experimental conditions**
**Li et al.** ***Phys. Chem. Chem. Phys.*** **2010**	75 nm Ag, 55 nm Au and Au@Pt (1.4 nm Pt) NPs, used to coat smooth electrodes, and dried in vacuum	SERS spectra collected at low and high resolutions	Under applied potential range: -0.4 to -1.9 V *vs.* SCE<br>Electrolyte: 0.1 M $NaClO_4$
**Fleischmann et al. J.** ***Electroanal. Chem. Interfacial Electrochem.*** **1981**	5 mm diameter polycrystalline Ag rod, polished to a mirror finish<br>And roughened Ag electrode using cyclic potential ramp up to +0.3 V at 5 mV s^{-1}	Raman / SERS: 514.5 nm, <100 mW<br><br>Surface spectra obtained by pushing electrode against cell window	Under applied potential range: -0.2 to -0.6 V *vs.* SCE<br>Electrolyte: 1-4 M KCl, 1 M KBr and 1 M KI
**Tian et al.** ***Electrochim Acta*** **1994**	Ag electrode, chemically polished then roughened by oxidation reduction cycle	SERS: 514.5 nm, ~100 mW	Under applied potential range: -0.2 to -2.0 V vs. SCE<br>Electrolyte: 8 M $NaClO_4$
**Tian et al.** ***J. Chem. Soc., Faraday Trans.*** **1996**	Single-crystalline Ag and polycrystalline Pt discs, mechanically/ chemically polished then roughened by oxidation reduction cycle	SERS: 514.5 nm, ~30 mW<br><br>Using thing layer cell and difference technique to subtract bulk contributions	Under applied potential range: -0.9 to -1.7 V vs. SCE<br>Electrolyte: 0.1 M $NaClO_4$, also 7 M $NaClO_4$, 6.5 M NaBr, 1-7 M NaI
**Wang at al.** ***Nature*** **2021**	Atomically flat Pd single-crystal (Pd(hkl)) surface	In situ Raman spectroscopy (SHINERS), 637.8 nm<br><br>Ultra-thin (50 µm) solution to minimise effects from bulk	Under applied potential range: 0.3 to -1.1 V *vs.* RHE<br>Electrolyte: 0.1, 1 and 8 M $NaClO_4$ (pH 11)
**Pettinger et al.** ***J. Chem. Phys.*** **1981**	Ag electrodes, mechanically polished, then etched in 10% ammonia/ H_2O_2 solution	SERS: 530.9 nm, ~ 1 W	Under applied potential range: -0.3 V vs. Ag/AgCl electrode<br>Electrolyte: 8 M NaCl or 10 M NaBr

Study	Substrate	Measurement technique	Experimental conditions
**Garcia-Araez et al.** *J. Phys. Chem. C* 2012	Gold thin layers deposited on silicon prism by electron beam evaporation at 0.1 and 1 Å/s, composed of 47 ± 11 nm or 27 ± 8 nm NPs	Surface-enhanced infrared absorption spectroscopy	Under applied potential range:<br>0.1 to 1.1 V *vs.* RHE<br>Electrolyte: 0.1 M $NaClO_4$
**Tatarkhanov et al.** *J. Am. Chem. Soc.* 2009	Pd(111) and Ru(0001) surfaces, prepared using cycles of ion bombardment, using noble gases (Ar, Ne) and flashing to 1100 or 1300 K	Scanning tunnelling microscopy	Ultra-high vacuum<br>Temperature range: 40-130 K, water adsorbed below 100 K
**Zhao et al.** *Appl. Surf. Sci.* 2020	20 monolayer Cd(0001) thin film grown on Si (111)-7×7	Scanning tunnelling microscopy	Ultra-high vacuum: $< 1.5 \times 10^{-10}$ Torr<br>Temperature: 4.6 or 78 K
**Dong et al.** *ACS Nano* 2018	Au(111) surface, prepared by cycles of sputtering and annealing	Scanning tunnelling microscopy, using tip functionalised with water or -OH group	Ultra-high vacuum: $< 3.0 \times 10^{-11}$ Torr<br>Temperature: 19 K
**Otto et al.** *Phys. Chem. Chem. Phys.* 2014	N/A	Raman: 532 nm	Gas mixtures of water with He, Ne/He or Ar/He expanded into Al chamber<br>Nozzle temperature: 25-120°C, saturator temperature: 10-22°C and stagnation pressure: 0.7-1.2 bar

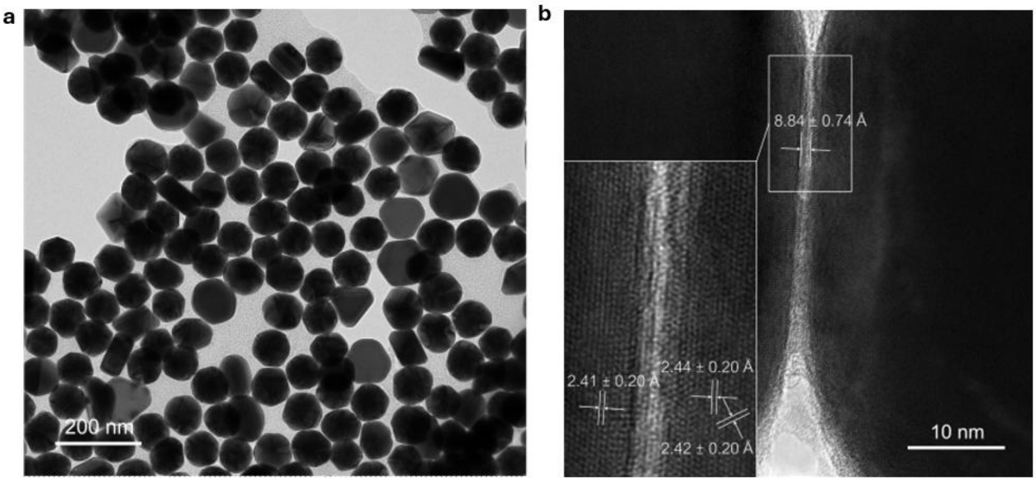

Figure S1. TEM images of (**a**) free-standing MLagg using commercial 80 nm AuNPs, (**b**) giving 0.9 nm nanogaps.

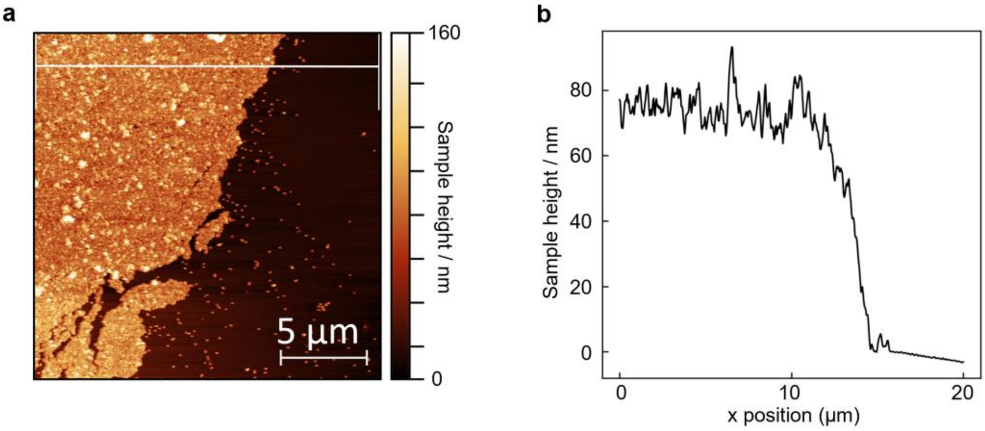

Figure S2. Atomic Force Microscopy (AFM) of a MLagg prepared with 80 nm AuNP. (**a**) AFM images of MLagg film near the edge, showing close-packed nanoparticles are mostly one monolayer high, as verified in (**b**) line section across the white line in (**a**). AuNP diameter is 80 nm.

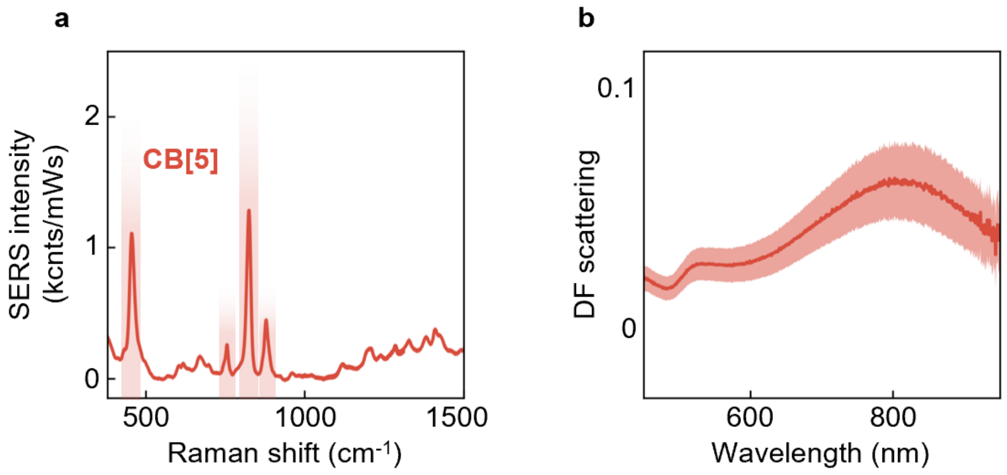

Figure S3. SERS and Dark field (DF) scattering of an MLagg prepared with 80 nm AuNPs. Map-scan SERS and DF measurements were taken over a 2500 μm^2 region on the sample (11×11 point grid, with 50 μm spacing), with a spectrum collected at each point on the grid. (**a**) Averaged SERS spectrum, with standard deviation shaded (barely visible either side of average curve). The relative standard deviation (RSD) of this sample is 4.8%, calculated from the peak height of the 826 cm^{-1} CB[5] peak, demonstrating that the MLagg sample is highly uniform. (**b**) Averaged DF scattering spectrum on the same positions, with standard deviation shaded, showing more variability but very similar spectral peak positions.

Supplementary Note 1: MLagg structure

MLaggs dry as close-packed arrays of 1-2 monolayers. Despite some inherent disorder in the structure (due to missing NPs, reduced coordination, and random in-plane close-packing orientation), the precision 0.9 nm gap spacing between adjacent NPs is set by the rigid CB[5] spacer molecules. This has previously been verified using spectroscopy on individual nanogaps[1] and ensures the plasmons in neighboring gaps have the same resonance wavelength and interact efficiently to give excellent light input/output coupling.

TEMs of free-standing 80 nm AuNP MLaggs directly resolve the 0.9 nm gaps between NPs (Figure S1). The continuous close-packed structure can also be seen using AFM on an 80 nm AuNP MLagg (Figure S2a). The MLagg is seen to be mainly monolayer, with very small regions of two monolayers (brighter regions). A line scan (Figure S2b) across the edge of the MLagg shows the 80 nm step in height from the substrate to the MLagg, corresponding to a single monolayer of 80 nm AuNPs.

Optical spectroscopy can give information over wider sample regions, and probe the CB[5] scaffolded nanogaps using scattering spectroscopy to show the plasmon resonance peak. The resonance wavelength is dependent on the nanogap width, and statistics over many individual nanogaps reveals a gap size of d = 0.9 ± 0.05 nm.[1]

Similarly, in MLaggs, where many nanogaps are combined, scattering spectroscopy gives a broad NIR peak from the coupled modes which allows us to track differences across large sample areas.[2,3]

By tracking both SERS intensity and the coupled resonance mode across a large MLagg region (2500 μm^2), uniformity can be demonstrated. The relative standard deviation (RSD), calculated from the peak height of the 826 cm^{-1} CB[5] peak, for a representative MLagg sample of 80 nm AuNP is 4.8% (Figure S3a). This low RSD is consistent across MLagg applications.[2–4] The dark-field (DF) scattering mode is also consistent across the sample (Figure S3b). Shifts in the DF resonance position to higher wavelengths can indicate two monolayer regions of the sample.[2]

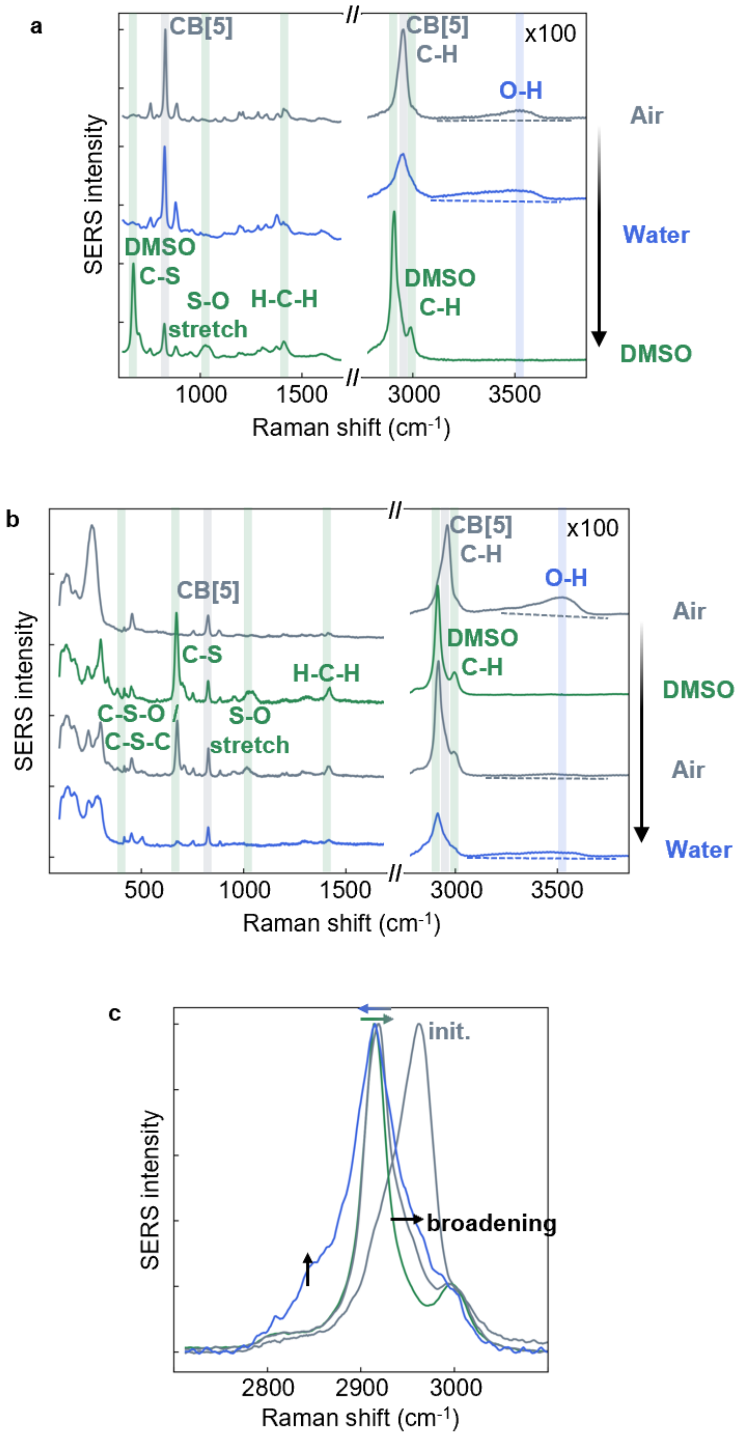

Figure S4. MLagg low and high wavenumber SERS in air, water and DMSO. (**a**) SERS in air, water and DMSO showing the differences in water hydrogen bonding network and exclusion by DMSO. (**b,c**) DMSO persists in the MLagg nanogaps even after drying and returning to water and causes permanent changes in the O-H and CB[5] C-H SERS peaks.

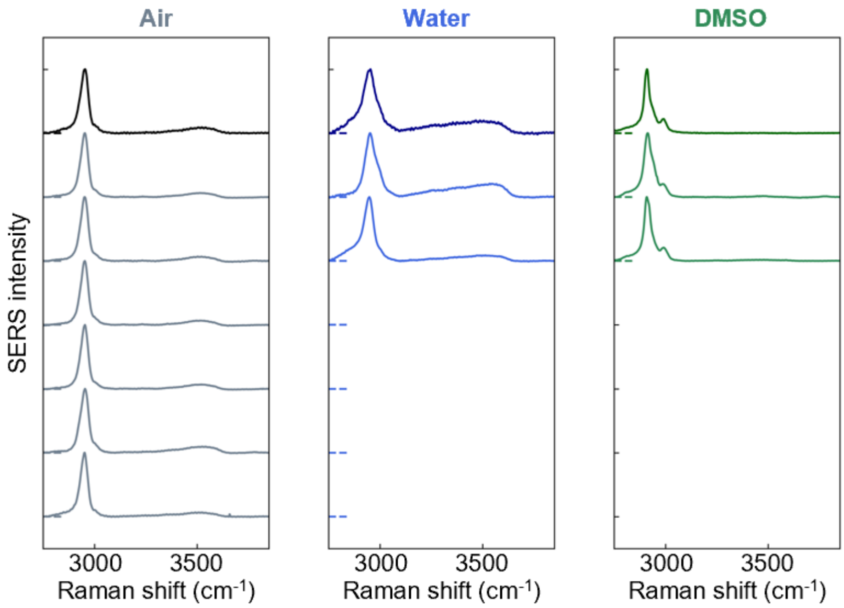

Figure S5. Repeat SERS data for MLaggs in air, water, and DMSO. SERS spectra for different CB[5] MLagg samples in air, or after 30 minute immersion in DI water, or in DMSO, show repeatability of measurements. Darker spectra (top) are data in Figure 1b.

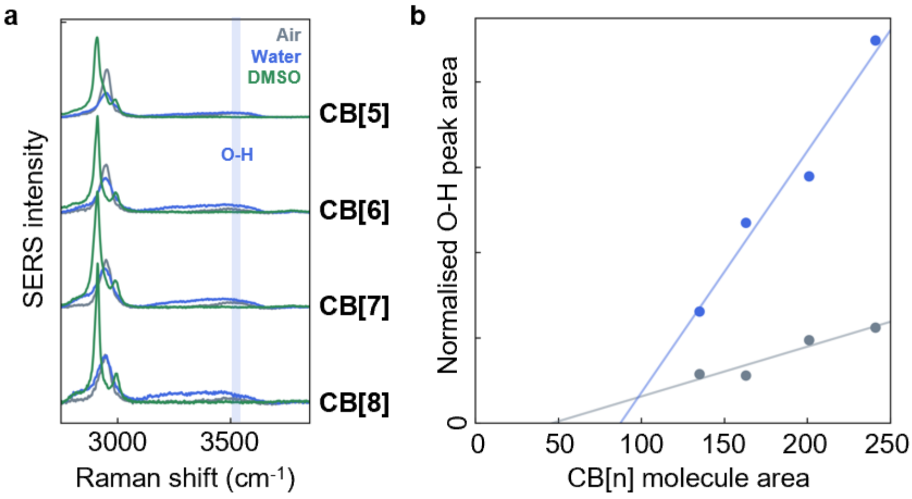

Figure S6. CB[n] scaffolded MLaggs SERS in air, water and DMSO. (**a**) SERS in air, water and DMSO for CB[5-8] scaffolds showing a similar effect on the O-H and C-H peaks for all CB[n]. (**b**) Plots of the normalized O-H peak area against CB[n] molecule area, showing a correlation between the water molecules present in the gap and the CB[n] size.

Supplementary Note 2: O-H/C-H CB[n] peak normalization

An approximate normalization to compare the water peaks for CB[5-8] was made by taking into account the C-H bonds per CB[n] molecules (C-H peak area/[n]) and the CB[n] per area of AuNP (1/CB[n] area). CB[n] area taken from literature[5].

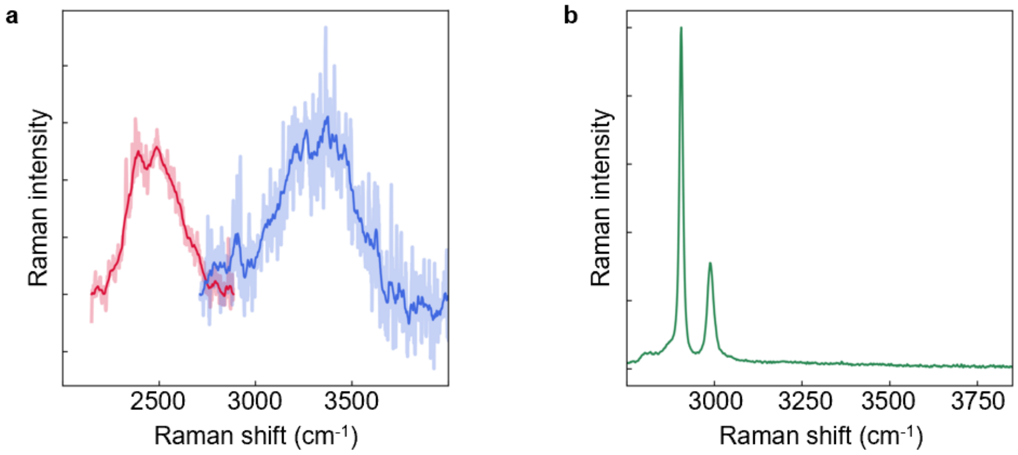

Figure S7. Raman of water and DMSO. (**a**) Raman of bulk H_2O (blue) and D_2O (red), with gaussian smoothed spectra in darker colours. (**b**) Raman of bulk DMSO.

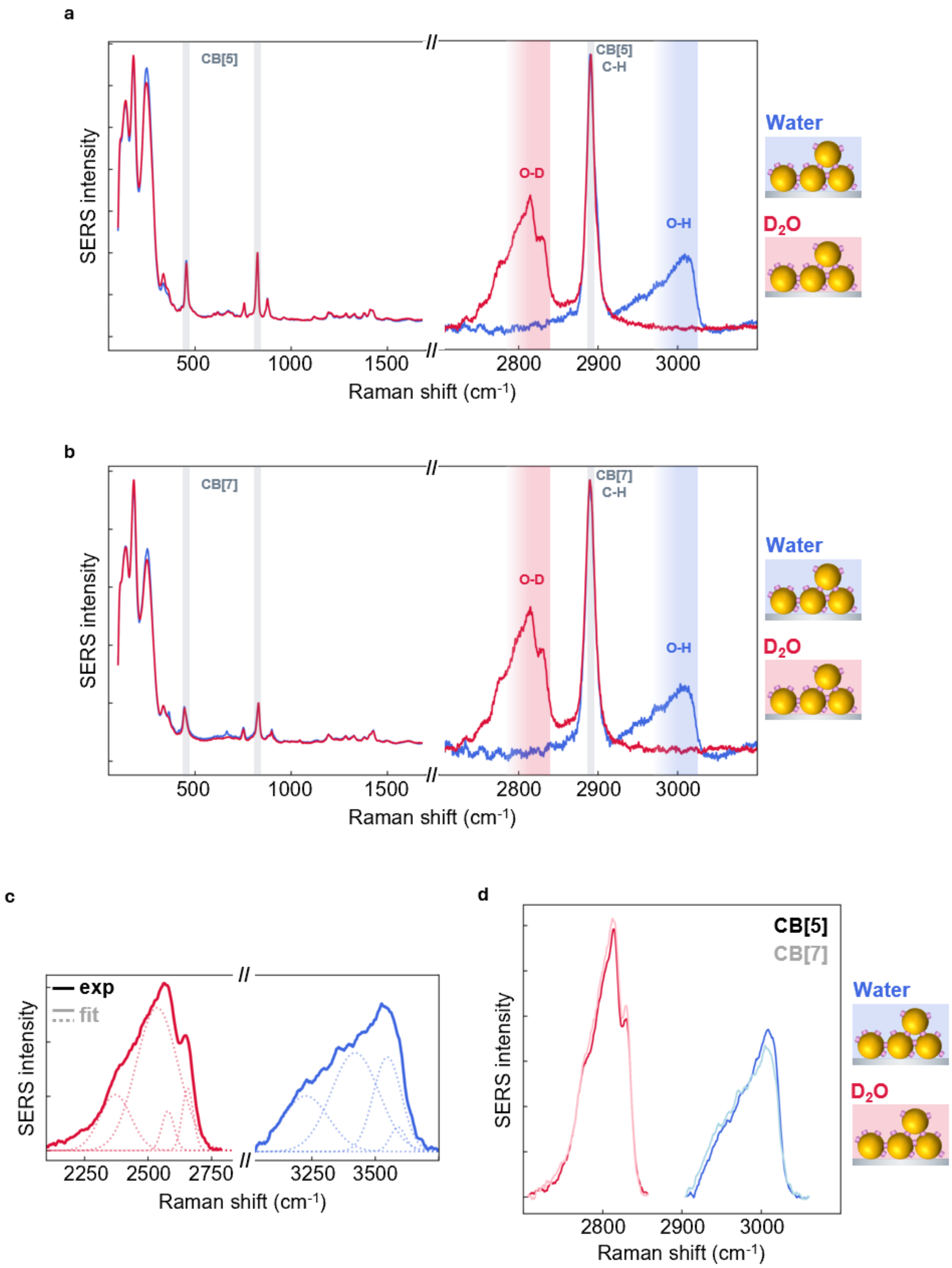

Figure S8. High and low wavenumber SERS of MLaggs scaffolded in aqueous and deuterated solutions. (**a**) SERS of CB[5] scaffolded MLaggs, showing the small CB[5] peak shifts and O-H and O-D peaks. (**b**) Corresponding SERS spectra for CB[7] scaffolded MLaggs. (**c**) Four peak fit of CB[7] MLagg O-H and O-D, with gaussian peaks in the same positions and with similar intensities as for CB[5]. (**d**) Overlaid O-H and O-D peaks for CB[5] and CB[7] MLaggs.

Supplementary Note 3: Protocol for fitting dimer peaks

To remove any excess background from the SERS, a linear background was first fit between the minima on either side of water or D_2O lines. Gaussian peaks were then fit with peak widths σ restricted between 20-100 cm^{-1}.

For the H_2O spectrum, peak positions were constrained based on previously reported peak positions: 3250 cm^{-1}, 3600 cm^{-1}, and an additional one or two peaks in the range 3000-3500 cm^{-1} (since [6] uses 3280, 3370, 3466, 3605 cm^{-1}, [7] uses 3300, 3450, 3600 cm-1, and [8] uses 3250, 3450, 3600 cm^{-1}). Peak fits are thus confined (within 30 cm^{-1}) to 3250 cm^{-1}, 3450 cm^{-1}, 3600 cm^{-1}, plus a fourth intermediate peak at 3545 cm^{-1}. This fits well, but as 12 variables are fitted, there is some uncertainty in fitted peak positions as it is difficult to get unique convergence. We thus check each fit from different starting parameters, and ensure that results on different locations are in agreement.

For the D_2O spectrum, the O-D peaks are much sharper so that first estimates of peak positions can be easily extracted and then allowed to vary by 20 cm^{-1} during subsequent fitting. For the CB[7] MLaggs, H_2O and D_2O peaks are fit using the peak positions found for CB[5] MLaggs (constrained to be within 5 cm^{-1} of CB[5] peak positions).

R^2 values were calculated for all fits:
CB[5]H_2O = 0.999, CB[5] D_2O = 0.994
CB[7] H_2O = 0.998, CB[7] D_2O = 0.991

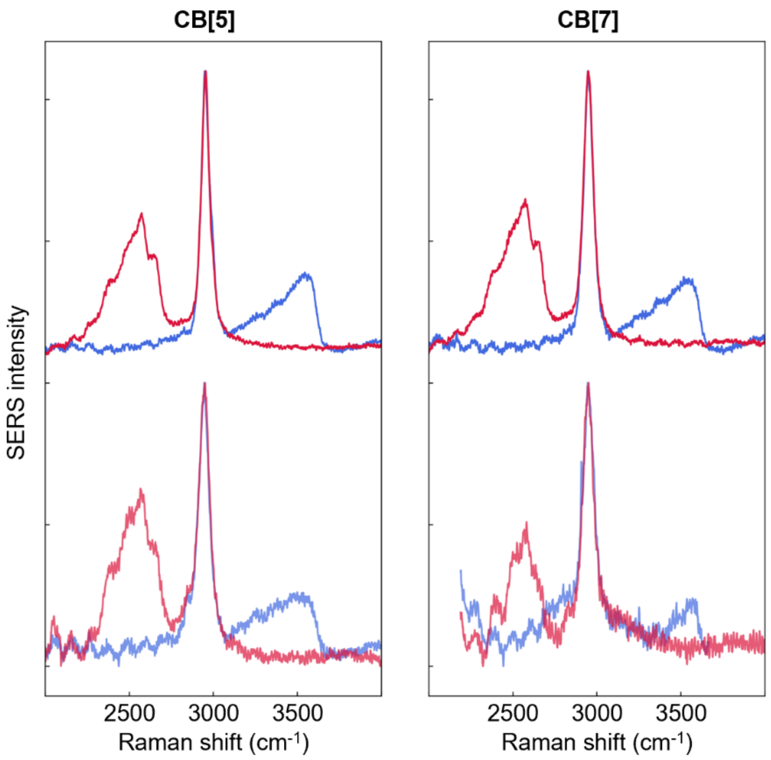

Figure S9. Repeat SERS data for MLaggs in scaffolded in aqueous and deuterated solutions. SERS spectra for different CB[5] and CB[7] MLagg samples re-scaffolded in aqueous (blue) and deuterated (red) solutions to show repeatability of measurements. Darker spectra are data in Figures 2 and S8.

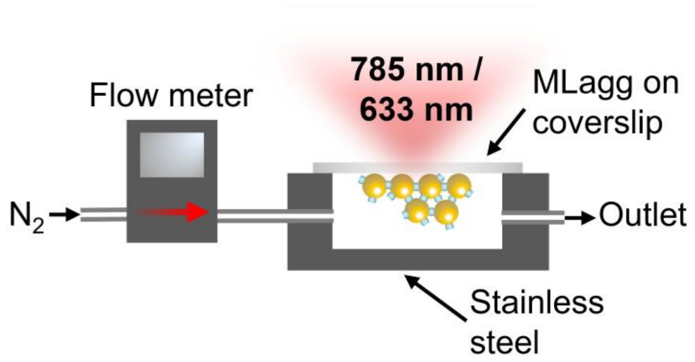

Figure S10. Experimental set-up for MLagg drying study. Schematic diagram of set-up for MLagg drying in N_2 flow.

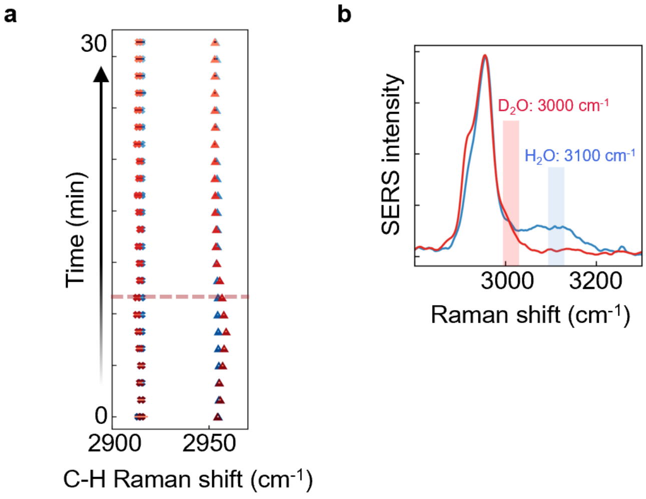

Figure S11. Changes in C-H peaks on drying. (**a**) Variation of the Raman shifts of the main C-H lines occurs over the same timescale as other C-H changes. (**b**) High wavenumber shoulder peaks are visible in both H_2O and D_2O at 3100 cm^{-1} and 3000 cm^{-1} respectively.

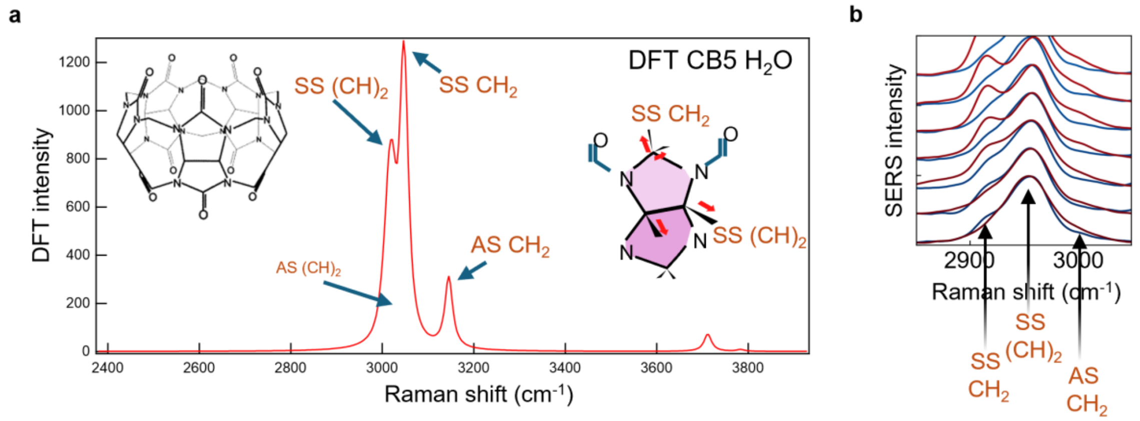

Figure S12. DFT of CB[5] C-H lines. (**a**) DFT of CB[5] containing cavity water, with C-H line split into four peaks corresponding to stretches of the CB[5] CH and CH_2. (**b**) Three of these peaks can be observed in the SERS spectra of the drying MLaggs.

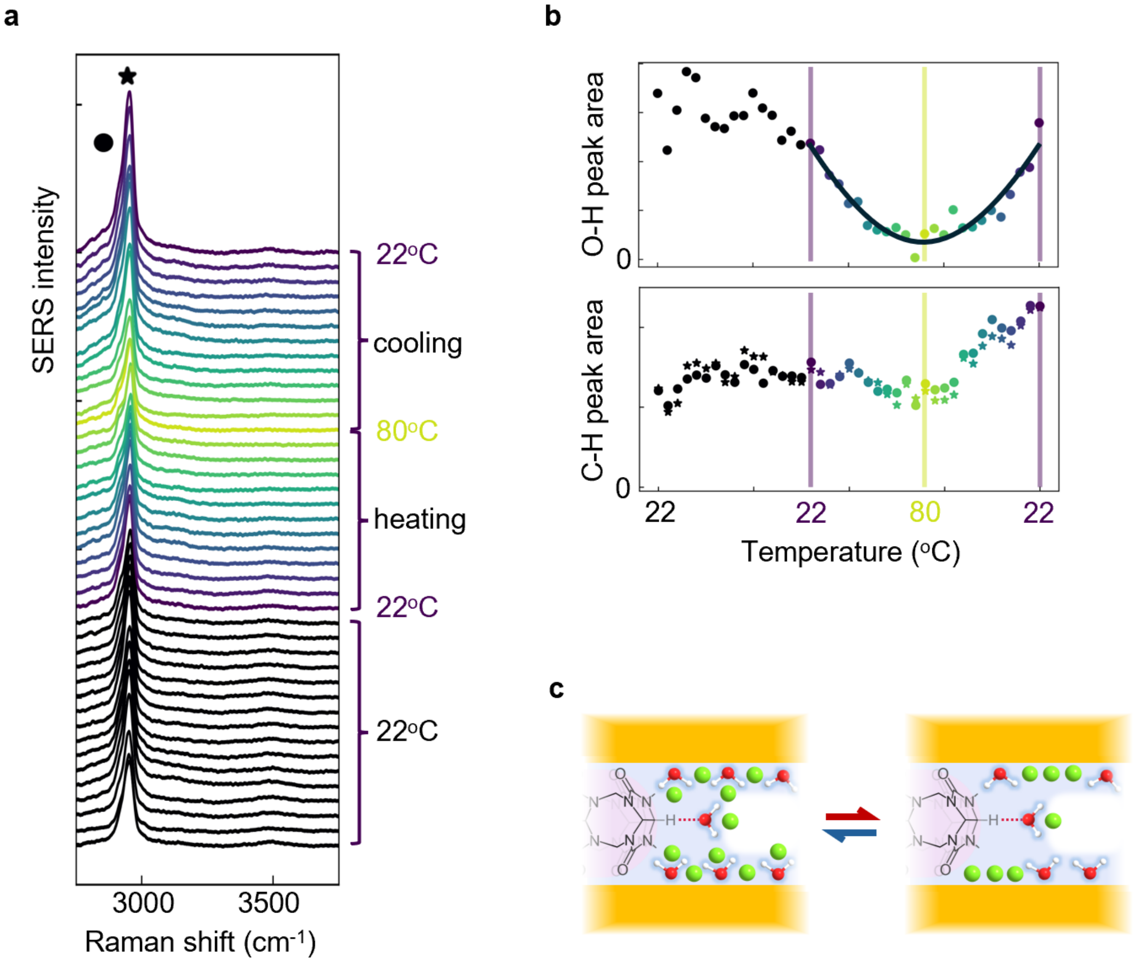

Figure S13. Heating and cooling. (**a**) SERS spectra taken as a dry CB[5]-scaffolded MLagg in 50 sccm N_2 flow is heated from room temperature (22°C) to 80°C and cooled. (**b**) Peak areas of the O-H and C-H lines as the sample was heated and cooled. (c) Possible arrangements of water and chloride (green) ions as the MLagg is heated and cooled.

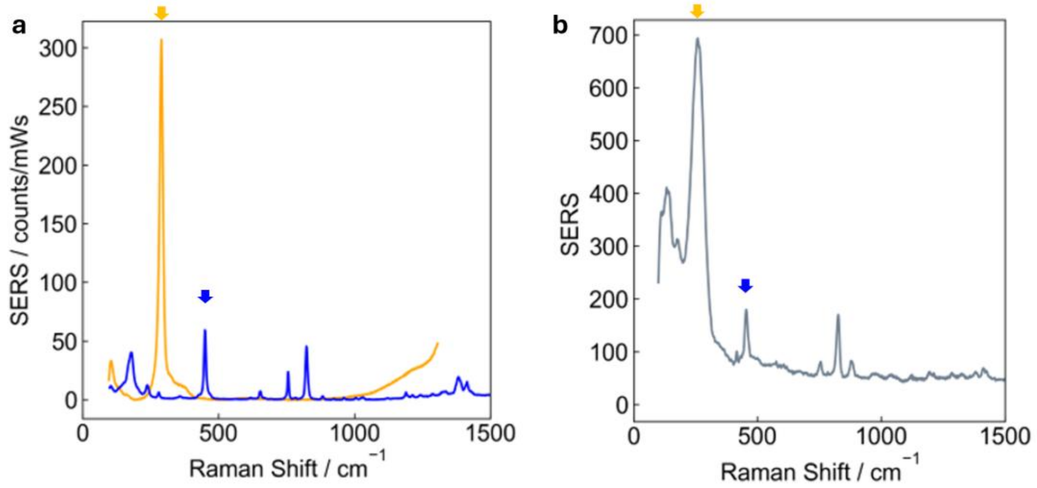

Figure S14. Ratio of CB[5] to Cl$^-$. (**a**) Powder Raman of AuCl (yellow) and CB[5] (blue) molecules, showing the ratio of signature peaks (arrows). (**b**) SERS of re-scaffolded MLagg in air (from Figure S3b) showing ratio of the same lines.

Supplementary Note 4: Estimation of CB[5] to Cl$^-$ ion ratio

We estimate the CB[5] to Cl$^-$ ion ratio from the Raman cross sections of peaks in powder Raman (of AuCl and CB[5]) and MLaggs. From the ratio of signature peaks (Figure S14), we estimate 5 Cl$^-$ ions per CB[5] molecule in the nanogaps. Previous work ([3] SI Figure S6, and Nature Chem. under review) shows that these Cl$^-$ are bound to the Au facets outside the CB[5], and they can be reversibly stripped off during the potential cycling process.

However, we expect that Cl$^-$ ions do not play a significant role in later electrochemical measurements as these take place in 1 M phosphate buffer.

Supplementary Note 5: Estimations of water binding enthalpy and entropy

Water peaks decrease by 100% x (40-5)/40 = 90%.

Bound water is in equilibrium with the surrounding environment. An open interface (rather than a nanogap) is assumed, but preliminary molecular dynamics shows this is reasonable even for confinement in gaps of ~1 nm.

Assuming a 1st order desorption process: $[H2O]_{aq}$ + BS = $[H2O]_{B}$, where BS is binding site concentration and $[H2O]_{aq}$ ~1 as in solution. For fixed binding site density, from the measured signals from bound water, R = $[H2O]_{B}$ at different temperatures (assumed to be at thermal equilibrium with the heating element), we can estimate the free energy change per molecule from heating from T_1 to T_2 as

$$dG_1 \; = \; k_B T_1 ln(c\, R_1)$$

$$dG_2 \; = \; k_B T_2 ln(c\, R_2)$$

So ratio $\frac{R_1}{R_2} = \frac{5}{40} = 0.125 \; = \exp\left(\frac{dG_1}{k_B T_1} - \frac{dG_2}{k_B T_2}\right) \sim \exp\left(dG\left(\frac{1}{k_B T_1} - \frac{1}{k_B T_2}\right)\right) \sim \exp\left(\frac{dG}{k_B} . \frac{dT}{T^2}\right)$

For dT =60K, we find the free energy change per molecule from heating, $dG \sim 280$ meV, which is a strong binding association.

To estimate the bound water surface coverage at 20°C, we use the equilibrium constant: $K = \frac{\theta}{1-\theta}$, for $\theta =$ fractional surface coverage[9], with state 1= 80°C, state 2 = 20°C. This gives

$$K = \exp\left(\frac{\Delta S}{k_B}\right) . \exp\left(-\frac{\Delta H}{k_B T}\right)$$

$$\frac{K_1}{K_2} = \exp\left[\Delta H\left(\frac{1}{k_B T_2} - \frac{1}{k_B T_1}\right)\right] \sim \exp\left[\frac{\Delta H}{k_B T}\frac{T_2 - T_1}{T}\right] \sim \frac{\theta_1}{\theta_2}\frac{1-\theta_2}{1-\theta_1}$$

So for surface coverage ratio $\theta_2 = R\theta_1$

$$\frac{\Delta H}{k_B T} \sim \frac{T}{T_2 - T_1}\ln\left[\frac{\theta_1}{\theta_2}\frac{1-\theta_2}{1-\theta_1}\right] \sim \frac{T}{T_2 - T_1}\ln\left[\frac{1}{R}\frac{1-R\theta_1}{1-\theta_1}\right] = \frac{T}{T_2 - T_1}\ln\left[\frac{1/R - \theta_1}{1-\theta_1}\right]$$

From the ratio, $R = 1/0.125 = 8$ and assuming a final coverage $\theta_1 \sim 0.1$, we find $\Delta\mathrm{H} \sim 300$ meV ($\sim 10\, k_B T_2$). Knowing K, we can estimate ΔS at 20°C:

$$\frac{\Delta S}{k_B} = \ln\left[\frac{\theta_2}{1-\theta_2}\right] + \frac{\Delta H}{k_B T_2}$$

Assuming that $\theta_2 = R\theta_1 \sim 0.8$, so $\ln\left(\frac{0.8}{1-0.8}\right) = 1.39$, this gives

$$\frac{\Delta S}{k_B} = 1.39 + \Delta H(k_B T_2) \sim 14$$

at room temperature, and $T_{bind} = \frac{dH}{dS} \sim 300$ K.

To check these values are reasonable, we compare the enthalpic part to electrostatic binding of polarised water with its image dipoles (water dipole p = 6.1 x 10^{-30} C.m), estimating a water molecule is 1.5 Å from the gold surface, so d=3 Å from its image dipole. The coulomb dipole-dipole energy,

$$U \; = \; 2\left(\frac{1}{4\pi\varepsilon_0\varepsilon}\right)\frac{p^2}{d^3} = 0.15 \text{ eV}$$

which is indeed similar to the above ΔH. Comparing the entropic part to the configurational entropy, $S = k \ln W$ we have $\frac{W_{\text{free}}}{W_{\text{bound}}} = \exp\frac{dS}{K} = e^{10} = 23000$. This is large, implying molecules are significantly constrained when bound to surface (for comparison $k_B T$ = 2.5 kJ/mol). This gives $TdS = 10\, k_B T = 25\,\text{kJ/mol}$, which is comparable to other calculated binding energies[10–12].

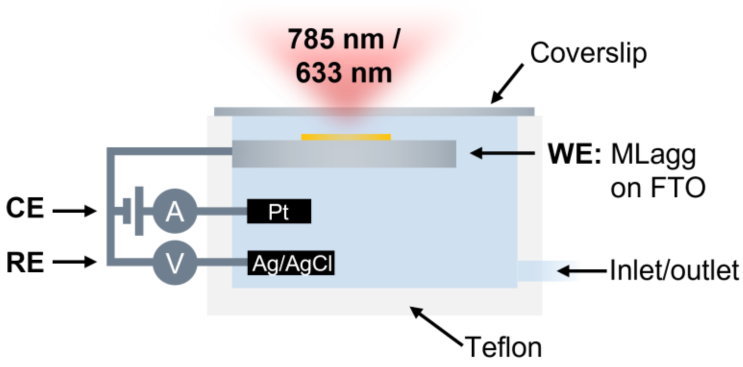

Figure S15. Experimental set-up for EC-SERS measurements. Cross-section of the three electrode electrochemical cell, with optical access to the MLagg through top glass coverslip. CE = counter electrode, RE = reference electrode and WE = working electrode.

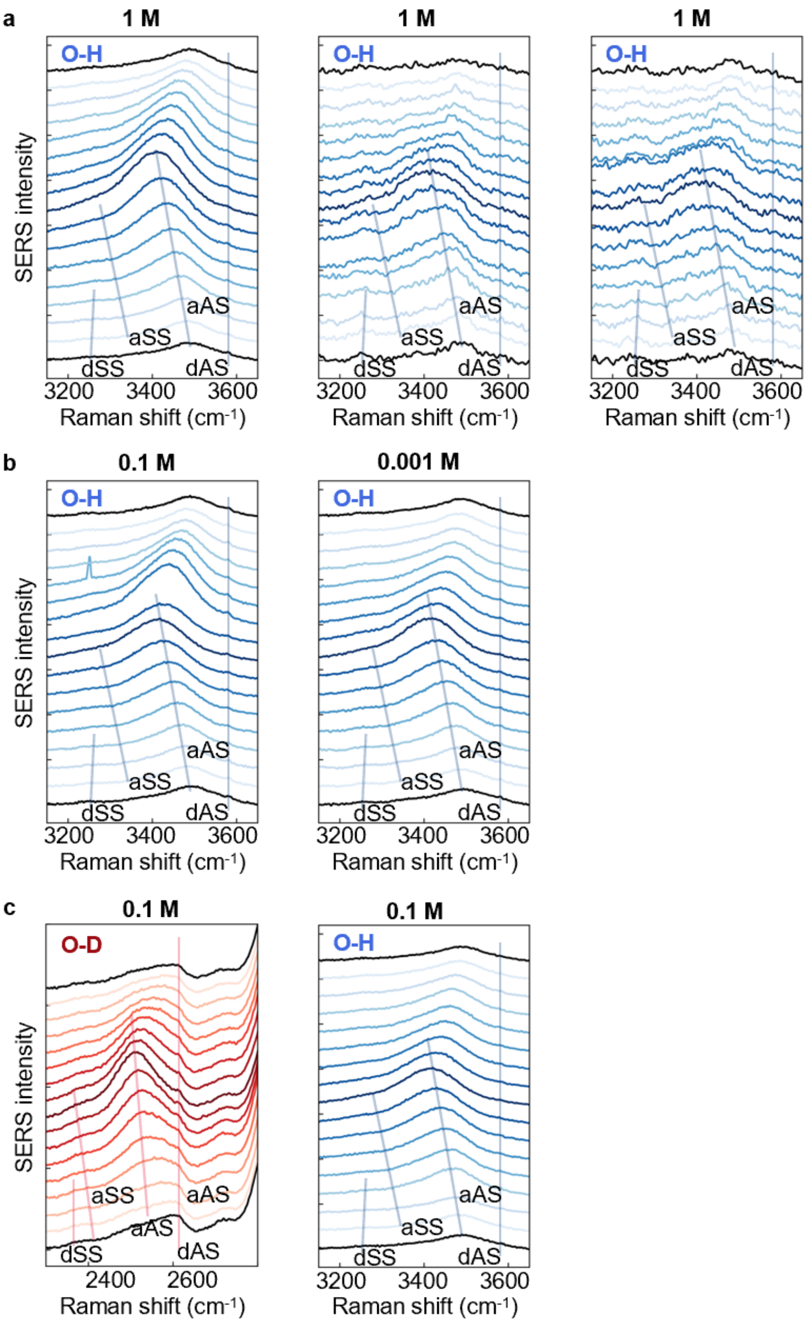

Figure S16. Repeat data for EC-SERS measurements. SERS spectra for CB[5] MLaggs applying potential from 0 V to -0.8 V and back in 0.1 V steps, for (**a**) 3 MLagg samples in 1 M phosphate buffer (repeats 2 and 3 at lower laser power), (**b**) MLaggs in 0.1 M and 0.001 M phosphate buffer and (**c**) MLaggs in 0.1 M perchlorate buffer. Between experiments, the position and shift of the four dimer peaks seen in the OH and OD spectra are highly consistent (lines).

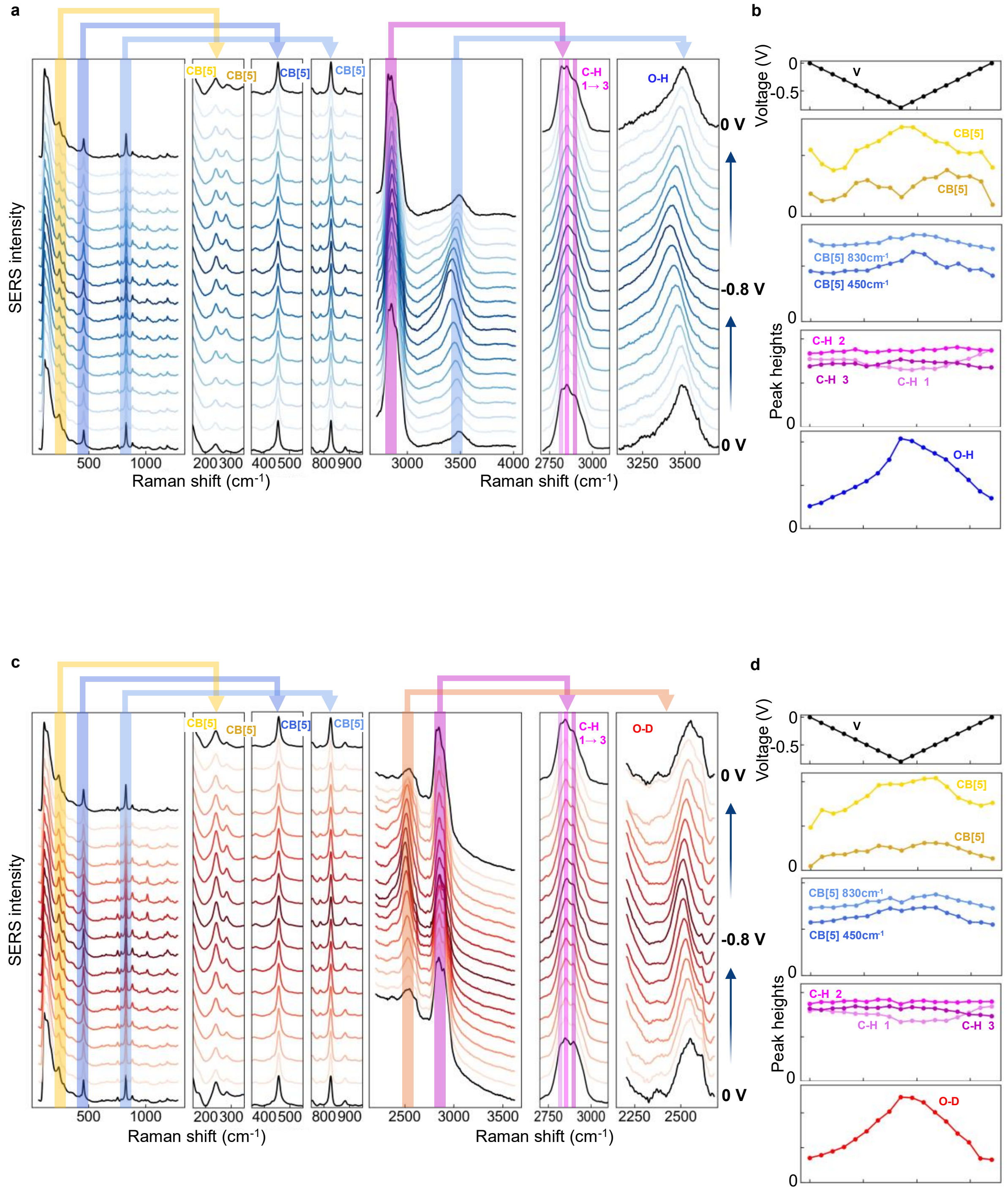

Figure S17. High and low wavenumber EC-SERS. SERS spectra for CB[5] MLaggs applying potential from 0 V to -0.8 V and back in 0.1 V steps, (**a**) in 1 M phosphate buffer with peak heights plotted (**b**), and (**c**) in 1M deuterated phosphate buffer with peak heights plotted in (**d**).

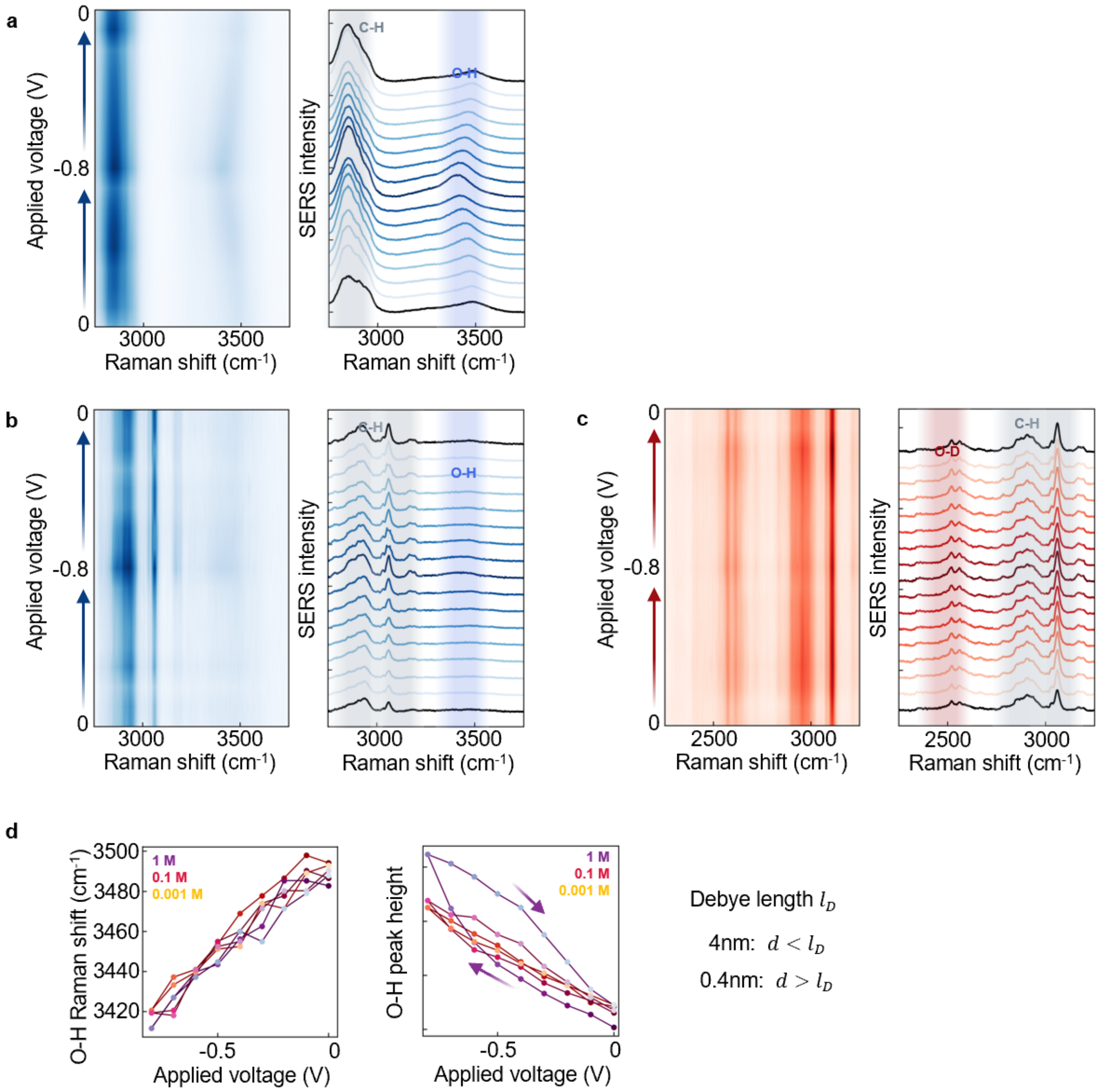

Figure S18. High and low wavenumber EC-SERS with CB[7] and BPT scaffolds. SERS spectra applying potential from 0 V to -0.8 V and back in 0.1 V steps in 1 M phosphate buffer, for (**a**) a CB[7] scaffolded MLagg, (**b**) a BPT scaffolded MLagg and (**c**) a BPT scaffolded MLagg in deuterated buffer. BPT excludes water from the gold surface. (**d**) O-H peak position and maximum height for CB[5] MLaggs in 1 M, 0.1 M and 0.001 M phosphate buffer.

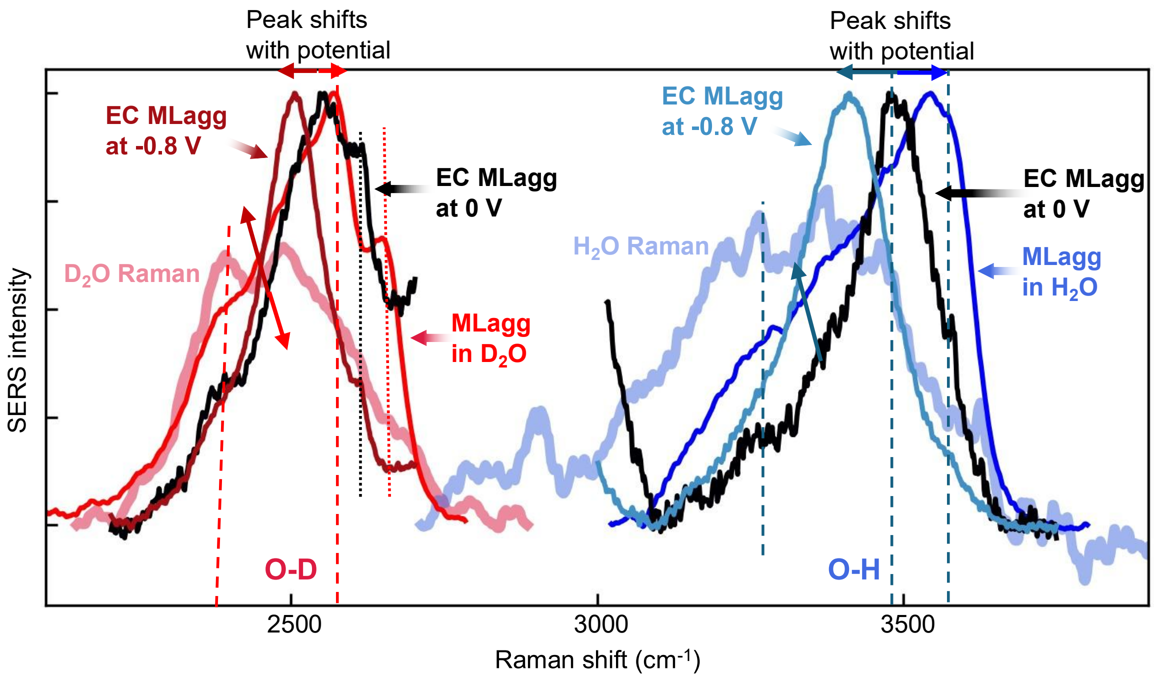

Figure S19. O-H and O-D in different samples. Comparison of O-H and O-D lines for the samples used throughout this work, showing the effects of bulk and surface water and the presence of ions on the peaks.

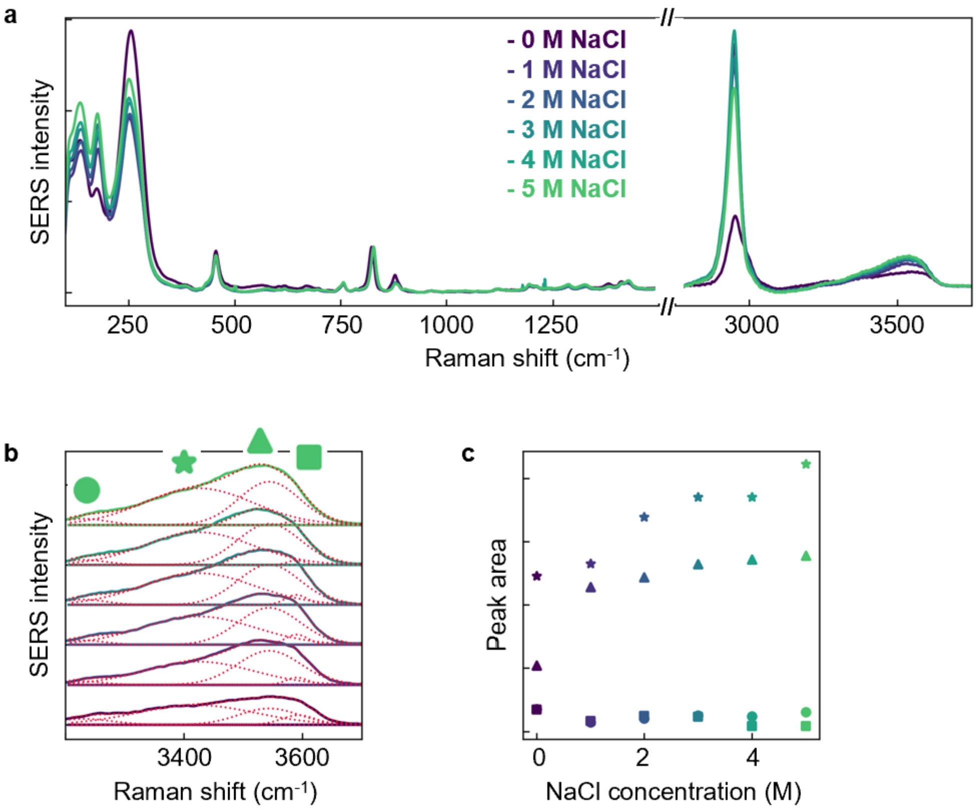

Figure S20. SERS in high NaCl concentrations. (**a**) SERS of a CB[5] MLagg in NaCl concentrations from 0-5 M. (**b,c**) 4-peak fit of the O-H SERS, and change in the peak area with increasing NaCl concentration (from bottom to top).

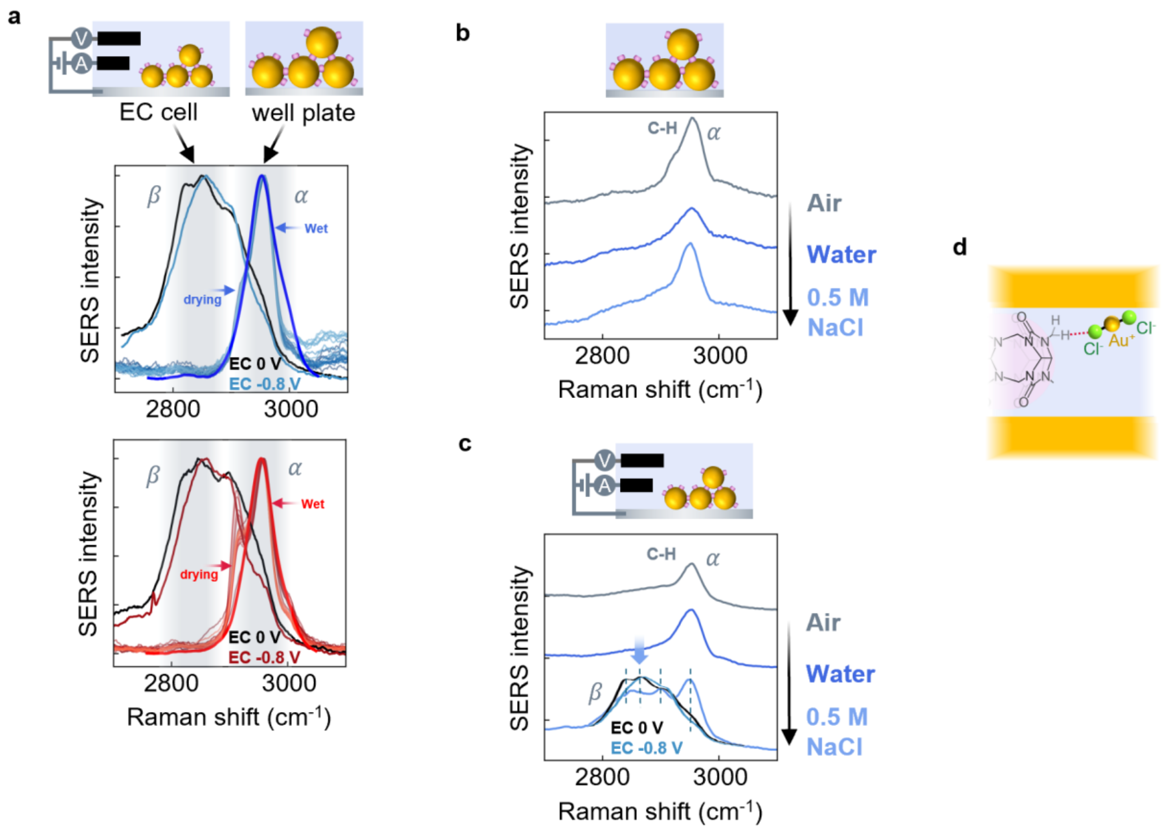

Figure S21. C-H in different samples. Comparison of CB[5] C-H lines for the samples used throughout this paper, showing the notable difference of the C-H lines in the EC cell. Two samples were immersed in water then 0.5 M NaCl, (**b**) in a well plate (**c**) and in the EC cell at OCP. The extra lower wavenumber C-H peaks appear when ions are present in the EC cell only. (**d**) Suggested effect of chloride ions present from the NaCl interacting with the C-H of the CB[5], in the presence of the reference and counter electrodes.

Supplementary Note 6: Choice of 785 nm and 633 nm laser wavelengths for Raman and SERS measurements

To collect spectra in the fingerprint and low Raman shift regions (100 – 1800 cm-1), a 785 nm excitation laser was used, while a 633 nm excitation laser was used for higher Raman shifts (2000 - 4000 cm^{-1}) as discussed below.

For MLagg structures of 80 nm nanoparticles with 0.9 nm CB[5] defined interparticle gaps, the plasmon resonance wavelength occurs at ~800 nm and hence SERS enhancement can be optimised for 785 nm laser excitation.[2] For this reason, we use a 785 nm excitation laser for measurements in the lower Raman wavenumber ranges (100 - 1800 cm^{-1}).

However, for higher Raman shifts (2000 - 4000 cm-1), the SERS light collected by the spectrometer CCD would have wavelength >1000 nm. At such wavelengths, the quantum efficiency of a silicon CCD is poor and results in reduced intensity of the SERS spectrum collected. By using a 633 nm excitation laser for these measurements, our detected light is at shorter wavelengths, where the CCD has better detection efficiency, but still has relatively good coupling to the MLagg substrate.

In both cases, laser power and integration time was chosen to provide good SNR in the SERS spectrum, while minimising sample damage (no loss of SERS intensity with repeat measurements).

References:

(1) De Nijs, B.; Bowman, R. W.; Herrmann, L. O.; Benz, F.; Barrow, S. J.; Mertens, J.; Sigle, D. O.; Chikkaraddy, R.; Eiden, A.; Ferrari, A.; Scherman, O. A.; Baumberg, J. J. Unfolding the Contents of Sub-Nm Plasmonic Gaps Using Normalising Plasmon Resonance Spectroscopy. *Faraday Discuss* **2015**, *178* (0), 185–193. https://doi.org/10.1039/C4FD00195H.

(2) Grys, D. B.; Niihori, M.; Arul, R.; Sibug-Torres, S. M.; Wyatt, E. W.; de Nijs, B.; Baumberg, J. J. Controlling Atomic-Scale Restructuring and Cleaning of Gold Nanogap Multilayers for Surface-Enhanced Raman Scattering Sensing. *ACS Sens* **2023**, *8* (7), 2879–2888. https://doi.org/10.1021/ACSSENSORS.3C00967.

(3) Sibug-Torres, S. M.; Grys, D. B.; Kang, G.; Niihori, M.; Wyatt, E.; Spiesshofer, N.; Ruane, A.; de Nijs, B.; Baumberg, J. J. In Situ Electrochemical Regeneration of Nanogap Hotspots for Continuously Reusable Ultrathin SERS Sensors. *Nature Communications 2024 15:1* **2024**, *15* (1), 1–13. https://doi.org/10.1038/s41467-024-46097-y.

(4) Niihori, M.; Földes, T.; Readman, C. A.; Arul, R.; Grys, D. B.; Nijs, B. de; Rosta, E.; Baumberg, J. J. SERS Sensing of Dopamine with Fe(III)-sensitized Nanogaps in Recleanable AuNP Monolayer Films. *Small* **2023**, *19* (48), 2302531. https://doi.org/10.1002/smll.202302531.

(5) Barrow, S. J.; Kasera, S.; Rowland, M. J.; Del Barrio, J.; Scherman, O. A. Cucurbituril-Based Molecular Recognition. *Chem Rev* **2015**, *115* (22), 12320–12406. https://doi.org/10.1021/ACS.CHEMREV.5B00341/ASSET/IMAGES/MEDIUM/CR-2015-00341F_0031.GIF.

(6) Hwang, A. Y.; Chikkaraddy, R.; Grys, D. B.; Scherman, O. A.; Baumberg, J. J.; de Nijs, B. Tracking Water Dimers in Ambient Nanocapsules by Vibrational Spectroscopy. *Proc Natl Acad Sci U S A* **2022**, *119* (49). https://doi.org/10.1073/PNAS.2212497119/-/DCSUPPLEMENTAL.

(7) Li, C.-Y.; Le, J.-B.; Wang, Y.-H.; Chen, S.; Yang, Z.-L.; Li, J.-F.; Cheng, J.; Tian, Z.-Q. In Situ Probing Electrified Interfacial Water Structures at Atomically Flat Surfaces. *Nat Mater* **2019**, *18* (7), 697–701. https://doi.org/10.1038/s41563-019-0356-x.

(8) Wang, Y. H.; Jin, X.; Xue, M.; Cao, M. F.; Xu, F.; Lin, G. X.; Le, J. B.; Yang, W. M.; Yang, Z. L.; Cao, Y.; Zhou, Y.; Cai, W.; Zhang, Z.; Cheng, J.; Guo, W.; Li, J. F. Characterizing Surface-Confined Interfacial Water at Graphene Surface by in Situ Raman Spectroscopy. *Joule* **2023**, *7* (7), 1652–1662. https://doi.org/10.1016/J.JOULE.2023.06.008.

(9) Douglas, J. F.; Freed, K. F.; Dudowicz, J.; Douglas, J. F. Lattice Theory of Competitive Binding: Influence of van Der Waals Interactions on Molecular Binding and Adsorption to a Solid Substrate from Binary Liquid Mixtures. *J Chem Phys* **2018**, *149*, 44704. https://doi.org/10.1063/1.5040105.

(10) Dunitz, J. D. The Entropic Cost of Bound Water in Crystals and Biomolecules. *Science (1979)* **1994**, *264* (5159), 670.

(11) Huggins, D. J. Quantifying the Entropy of Binding for Water Molecules in Protein Cavities by Computing Correlations. *Biophys J* **2015**, *108*, 928–936. https://doi.org/10.1016/j.bpj.2014.12.035.

(12) Fleck, M.; Zagrovic, B. Configurational Entropy Components and Their Contribution to Biomolecular Complex Formation. *J Chem Theory Comput* **2019**, *15* (6). https://doi.org/10.1021/acs.jctc.8b01254.
